# Surface Immuno-Functionalisation for the Capture and Detection of *Vibrio* Species in the Marine Environment: A New Management Tool for Industrial Facilities

**DOI:** 10.1371/journal.pone.0108387

**Published:** 2014-10-13

**Authors:** Olivier F. Laczka, Maurizio Labbate, Justin R. Seymour, David G. Bourne, Stewart S. Fielder, Martina A. Doblin

**Affiliations:** 1 Plant Functional Biology and Climate Change Cluster (C3), University of Technology, Sydney, NSW, Australia; 2 The ithree institute, University of Technology, Sydney, NSW, Australia; 3 Australian Institute for Marine Science, Townsville, QLD, Australia; 4 Port Stephens Fisheries Institute, New South Wales Department of Primary Industries, Taylors Beach, NSW, Australia; Universidade Federal do Rio de Janeiro, Brazil

## Abstract

Bacteria from the genus *Vibrio* are a common and environmentally important group of bacteria within coastal environments and include species pathogenic to aquaculture organisms. Their distribution and abundance are linked to specific environmental parameters, including temperature, salinity and nutrient enrichment. Accurate and efficient detection of Vibrios in environmental samples provides a potential important indicator of overall ecosystem health while also allowing rapid management responses for species pathogenic to humans or species implicated in disease of economically important aquacultured fish and invertebrates. In this study, we developed a surface immuno-functionalisation protocol, based on an avidin-biotin type covalent binding strategy, allowing specific sandwich-type detection of bacteria from the *Vibrio* genus. The assay was optimized on 12 diverse *Vibrio* strains, including species that have implications for aquaculture industries, reaching detection limits between 7×10^3^ to 3×10^4^ cells mL^−1^. Current techniques for the detection of total Vibrios rely on laborious or inefficient analyses resulting in delayed management decisions. This work represents a novel approach for a rapid, accurate, sensitive and robust tool for quantifying Vibrios directly in industrial systems and in the environment, thereby facilitating rapid management responses.

## Introduction

Vibrios are a Gram-negative bacterial genus found in both tropical and temperate marine environments [1]–[3]. In recent years there has been growing interest in the dynamics of *Vibrio* populations, because many strains are pathogenic to humans and marine animals and represent a significant threat to the aquaculture industry and human health [4], [5]. A global estimate of disease losses to aquaculture by the World Bank in 1997 was approximately US$3 billion per annum with Vibrios playing a significant role [6]. There is evidence that *Vibrio*-associated diseases are increasing globally, including seafood-linked human poisonings [7], human wound infections through contact with contaminated waters [8], [9] along with diseases reported in corals, molluscs and fish [10]–[13]. These increases in *Vibrio* distribution and virulence have been linked to climate change [14] and other environmental perturbations associated with human activities [15]–[17]. Given the emerging threat of marine diseases and their potential to detrimentally impact the aquaculture industries, there is a growing need for establishing rapid, on-site detection techniques for pathogenic marine bacterial groups, including the Vibrios.

Current techniques for detecting Vibrios in the environment are focused on the detection of specific strains, such as *V. cholerae, V. vulnificus and V. parahaemolyticus*, known to affect economically important aquaculture species as well as human health. Approaches used to examine these bacteria rely on time-consuming procedures, including culturing or quantitative molecular biological approaches (e.g. quantitative PCR), often resulting in management decisions being made days after the collection of samples [18]–[20]. Tools incorporating biosensor technology which allow real-time quantitative assessment of *Vibrio* populations in environmental samples offer considerable advantages over well-established methods, including low analysis cost, relatively short time-to-result, high potential for miniaturisation, and the possibility of performing the measurements *in situ* without technical expertise. Biosensing devices also allow for online monitoring of water systems enabling the development of near real-time ecosystem and aquaculture species health and disease surveillance platforms.

Previous efforts to make *Vibrio* biosensors have generally focused on the detection of human pathogenic strains [21], [22]. This study develops and optimises a robust functionalisation protocol allowing the specific capture of total Vibrios in seawater samples using selected anti-*Vibrio* antibodies as the recognition elements. We describe the optimisation of a sandwich-type assay using the avidin-biotin affinity as the strategy for the immobilisation of the capture antibodies, and horse-radish peroxidase (HRP) as the label for the detection antibody. We show the assay to be robust with real samples obtained from mulloway fish larvae (*Argyrosomus japonicas*) rearing tanks and that the technique allows for rapid identification of *Vibrio* strains previously implicated as pathogens within aquaculture settings. This work represents a major step towards the development of a biosensor for the detection of Vibrios in aquaculture and natural settings and the management of aquaculture facilities.

## Materials and Methods

### Ethics statement

This study was carried out in strict accordance with the recommendations in “A Guide to Acceptable Procedures and Practices for Aquaculture and Fisheries Research” [23]. The protocol was approved by the Animal Care and Ethics Committee of the NSW Department of Primary Industries (Fisheries) (Permit Number: 93/1). Larvae were reared under optimal conditions as described in [24] and all efforts were made to minimize suffering.

No specific permission was required for this location and none of the field studies conducted for this study involved endangered or protected species.

### Bacterial strains and growth conditions

Bacterial strains used in this study are listed in Table 1. At least seven out of twelve *Vibrio* species included in this work have been described as potential aquaculture pathogens [28]. The different strains were grown at 26°C in marine broth 2216 medium (Difco) overnight. For the surface immuno-functionalisation protocol, fresh cultures were aliquoted into 1.5 ml eppendorf tubes, and centrifuged for 10 minutes at 14,000 g. The resultant supernatants were discarded and the pellets were retrieved and stored at −20°C. Prior to freezing, total viable counts of colony forming units (CFU) were determined by serially diluting and plating onto marine agar (2216 medium; Difco). Prior to experiments, frozen pellets were thawed to room temperature and reconstituted in phosphate buffered saline (PBS, Sigma-Aldrich) solution, to the desired cell concentration.

**Table 1:** Bacterial strains used in this study.

Species	Strain	Source location
*Vibrio rotiferianus*	DAT722	UTS^1^ [25]
*Vibrio alginolyticus*	12G1	UTS [26]
*Vibrio harveyi*	ATCC14126	ATCC^2^
*Vibrio natriegens*	C5	UTS
*Vibrio campbellii*	C7	UTS
*Vibrio parahaemolyticus*	C8	UTS
*Vibrio cholerae* (non 01/0139)	S10	UTS [27]
*Vibrio coralliilyticus*	BAA-450	ATCC
*Vibrio shiloi*	BAA-91	ATCC
*Vibrio splendidus*	33125	ATCC
*Vibrio ordalii*	33509	ATCC
*Vibrio tubiashii*	19109	ATCC
*Serratia marcescens*	BAA-632	ATCC

1 University of Technology, Sydney Culture Collection.

2 American Type Culture Collection.

Sterile filtered seawater collected from Iron Cove, Sydney (33 51′57.31″S/151°8′51.05″E) was used for the optimisation of the detection system. For obtaining a conservative estimate of *Vibrio* and total marine bacteria colony forming units (CFU) ml^−1^, triplicate 100 µl samples of seawater were plated on Thiosulfate-Citrate-Bile-Sucrose (TCBS) agar and marine agar respectively, incubated for 24 hours and CFUs counted [29]. Although TCBS underestimates viable *Vibrio* numbers, consistent results were obtained and two media were used (both Marine Agar and TCBS) for the counts.

### Affinity test and colorimetric detection of *Vibrio* strains using targeted polyclonal antibodies

Horseradish peroxidase (HRP) labelled anti-*Vibrio* rabbit polyclonal antibody (HRP-α*Vib* Pab) (Kirkegaard & Perry Laboratories Inc; Washington, D.C, US) was assessed for affinity against a range of *Vibrio* bacterial strains (Table 1) and checked for cross reaction with another non-*Vibrio* ubiquitous bacterium found in marine systems, *Serratia marcescens* [30]. The stability of the all the antibodies used in this study was given by the manufacturer and is 3 months at 4 degrees Celsius and 1 year at −20 degrees Celsius. No loss of activity was observed during the 12 month experimental period. Increasing serial concentrations of target *Vibrio* strains (from 1 to 10^9^ cells mL^−1^), *S. marcescens* (negative control) and the antibody manufacturer's positive control cell extracts were adsorbed onto the multiwell plate (Nunc maxisorb, Sigma-Aldrich) surfaces. The wells were then washed (3 washes with 400 µl of PBS (10 mM pH 7.4) containing 0.05% v/v Tween (PBST; Sigma-Aldrich)) and their surfaces blocked through addition of 200 µl bovine serum albumin (BSA 2% (w/v); Sigma-Aldrich) for 2 hours at 37°C. After another washing procedure, 100 µl of a 1/500 HRP-α*Vib* Pab solution was added to the wells (standard dilution recommended by the antibody supplier KPL, Inc.) for 1 hour at room temperature. The final washing step involved 3 washes with 400 µl of PBST, followed by 2 washes with 400 µl PBS. One hundred µL of 3,3′,5,5′-Tetramethylbenzidine (TMB; Sigma-Aldrich) HRP substrate was then introduced to each plate well and left to develop for 10 min at room temperature. The reaction was stopped with 50 µL of stop solution (Sigma-Aldrich), and colour development was recorded at A450 nm using a plate reader (Fluostar optima, BMG labtech Pty. Ltd.).

### Surface immuno-functionalisation and capture antibody immobilisation

**Figure 1:**
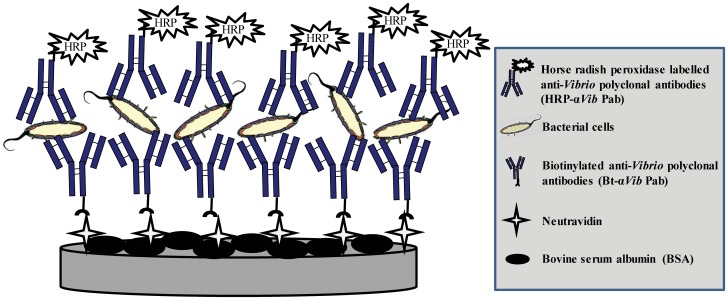
Sandwich-type format carried out in ELISA plate wells for the capture and detection of *Vibrio* cells. The well surface is coated with neutravidin and blocked with bovine serum albumine. The biotinylated anti-*vibrio* polyclonal antibody is added and bacterial cells are then captured and detected using an horse radish peroxidase linked anti-*vibrio* polyclonal antibody that can be detected colorimetrically using a spectrophotometer at 450 nm.

To obtain a surface that would specifically and efficiently capture *Vibrio* cells, we developed and optimised a protocol allowing for maximum coverage and optimum orientation of the antibodies (yielding maximum binding capacity). Any remaining uncovered surface was blocked with BSA to avoid any non-specific adsorption of cells to the surface (see Figure 1). Unless otherwise stated, all washing procedures consisted of two consecutive washes with PBST, followed by two washes with 400 µl of PBS. Briefly, 100 µl of neutravidin (20 µg mL^−1^ in PBS) was added to the Nunc maxisorb plate wells for 1 hour at 37°C followed by a washing step. These concentration and incubation conditions were optimised in a previous study [31]. Next, 100 µl of biotin labelled anti-*Vibrio* rabbit polyclonal antibody (Bt-α*Vib* Pab; KPL Inc; Washington, D.C, US) (20 µg mL^−1^ in PBS) was directly added to the well surface of the 96 well plates for 1 hour at 37°C. The wells were washed, blocked with 200 µl 2% BSA (w/v in PBS) for 2 hours at 37°C, and washed again. The negative controls consisted of wells functionalised with neutravidin and BSA but no antibody.

### Bacterial detection on an immuno-functionalised surface

Once the surface had been functionalised, the optimum conditions for bacterial cell capture were assessed by testing the appropriate contact times and the optimum amount of detection antibody (HRP-α*Vib* Pab). The wells functionalised with Bt-α*Vib* Pab or BSA only (negative controls for unspecific adsorption), were incubated with serial inoculations of between 1 to 1×10^9^ cells mL^−1^ bacteria, washed and incubated with 100 µl of HRP-α*Vib* Pab at room temperature (22±1°C) mimicking field conditions. For each condition tested, 3 replicates were carried out and the average and standard deviation of the absorbance values calculated. The assay limit of detection (LOD) was calculated as the mean of 10 blanks (assay carried out in the absence of bacteria) plus 3 times the standard deviation and expressed in cells mL^−1^. IC50 is the concentration in cells ml^−1^ generating 50% of the maximum assay absorbance (A) signal and was calculated as follows: (Amax – (Amax - Amin)/2). The assay sensitivity was calculated as the slope of the linear component of the curve and is expressed in absorbance units per unit of bacteria concentration (AU mL cell^−1^).

### Detection of *Vibrios* within mulloway fish larvae rearing tanks

A field test of the assay was conducted in the context of a mulloway rearing trial, where kaolin clay (Premium Clay, Boral Cements Blue Circle, Australia; 5 mg/L) and algal paste (Instant Algae, Reed Mariculture, USA; 3.5 mL/1000 L) were being evaluated as media for ‘greenwater’ in the rearing of mulloway (*Argyrosomus japonicus*) larvae. Fertilized eggs were sourced from captive mulloway broodstock at the Port Stephens Fisheries Institute (NSW, Australia) and hatched in 450 L incubators (salinity 32; 22°C). After hatching, the larvae were randomly stocked (4 larvae L^−1^) into six 2000 L experiment tanks filled with disinfected seawater (ozone 0.5–1.0 ppm; salinity 3232 ppt). The tanks had conical bottoms and were fitted with an upwelling manifold positioned at the base of the tank. Water flowed out of the tank through a 500 µm mesh-covered standpipe into a 200 L sump, where the water was returned to the tank via the manifold at 24 L min^−1^. In addition, 100% of the tank volume was exchanged daily with disinfected seawater. Artificial light was provided overhead from fluorescent lamps at 225–400 Lux with a photoperiod of 12 h light (0900 h to 2100 h) and 12 h dark [24] until 6 days post hatch, after which time photoperiod was increased to 18L:6D. Water temperature was 22.0±1°C.

Algae and clay treatments were added to the tanks every day after the daily water change. Fish larvae were fed rotifers (*Brachionus plicatilis*) enriched with Sparkle and Spresso (INVE Aquaculture, Belgium) according to manufacturer's instructions at a target density of 10 rotifers mL^−1^ from 2 days post hatch (dph). From 12 dph, larvae were fed *Artemia* enriched with Algamac 3050 (Aquafauna Biomarine, CA, USA) according to manufacturer's instructions. Surface skimmers were used during all experiments to remove oil debris from the water surface. Dissolved oxygen, water temperature, salinity and pH were measured daily using a multi-parameter water quality probe (Horiba U-10, Horiba Ltd., Japan). The trial was terminated at 26 dph and the larval survival rates were determined at harvesting.

At 14 dph, 50 ml sterile falcon tubes were used to collect water samples from each of the mulloway larvae rearing tanks (n = 3 samples per tank). Conservative estimates of Vibrios and marine bacteria were made using the TCBS culturing method and compared to estimates made using the ELISA detection test. 100 µL of fresh tank samples were added in triplicate to functionalised 96 well plates. A *V. parahaemolyticus* pellet (positive control) was resuspended in filtered seawater from the tanks water supply and used within the same plate in order to obtain a standard detection curve. The detection of captured *Vibrio* cells was then carried out using the developed ELISA method.

## Results and Discussion

### Antibody affinity to target *Vibrios*

**Figure 2:**
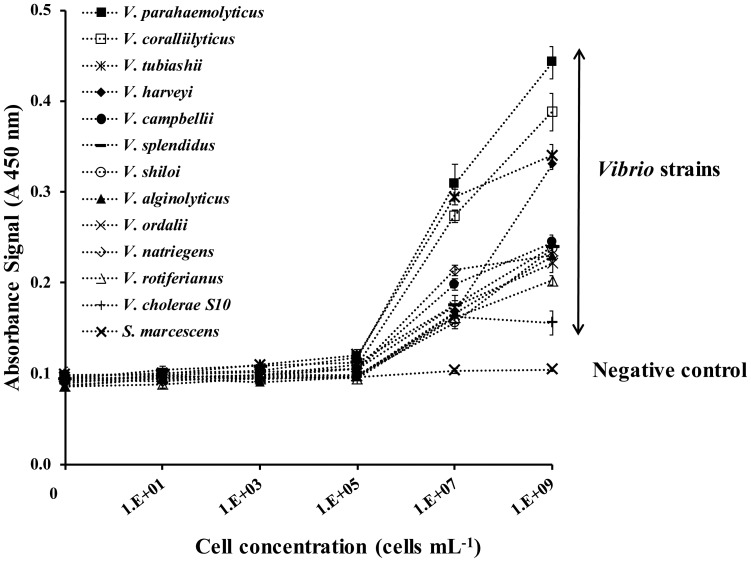
Antibody affinity for different *Vibrio* strains. Absorbance signals obtained after direct adsorption of bacterial strains to the well surface for one hour followed by detection using a 1/500 dilution of horseradish peroxidase anti-*Vibrio* antibody (HRP-α*Vib* Pab) incubated for one hour onto the surface.

Twelve temperate and tropical *Vibrio* strains were used to assess the specificity of the polyclonal antibodies used in this study. There was substantial variation in absorbance signal amplitudes which ranged between 0.08 and 0.45 (see Figure 2), demonstrating differences in the affinity of the antibody for the different *Vibrio* strains. However, the limit of detection (LOD) remained similar for all strains tested, ranging from 1×10^5^ to 5×10^5^ cells mL^−1^ (Table 2). *V. parahaemolyticus* and *V. tubiashii* showed a lower LOD than all other strains (1×10^5^ cells mL^−1^ and *V. parahaemolyticus* was chosen as the target for subsequent development and optimisation of the immuno-functionalised surface experiments due to its relevance for human health issues. No cross-reactivity or cross-binding was observed for the non-target control bacterium, *S. marcescens*.

**Table 2:** LOD, IC50 and sensitivity values obtained for each *Vibrio* strain with or without functionalising the plate surface.

STRAIN	Cells directly adsorbed on plate surface			Cells captured on functionalised surface		
	LOD (cells mL^−1^)	IC50 (cells mL^−1^)	Sensitivity (AU.mL cell^−1^)	LOD (cells mL^−1^)	IC50 (cells mL^−1^)	Sensitivity (AU.mL cell^−1^)
*Vibrio rotiferianus*	5.10^5^	8.10^6^	0.039	3.10^4^	5.10^6^	0.064
*Vibrio alginolyticus*	5.10^5^	8.10^6^	0.049	9.10^3^	4.10^5^	0.067
*Vibrio harveyi*	5.10^5^	8.10^6^	0.078	8.10^3^	2.10^6^	0.079
*Vibrio natriegens*	3.10^5^	10^6^	0.051	3.10^4^	10^6^	0.071
*Vibrio campbellii*	3.10^5^	3.10^6^	0.052	9.10^3^	5.10^5^	0.087
*Vibrio parahaemolyticus*	10^5^	2.10^6^	0.121	7.10^3^	2.10^5^	0.129
*Vibrio cholerae* S10	5.10^5^	8.10^5^	0.024	8.10^3^	2.10^5^	0.087
*Vibrio coralliilyticus*	3.10^5^	3.10^6^	0.103	8.10^3^	10^6^	0.128
*Vibrio shiloi*	5.10^5^	2.10^7^	0.047	8.10^3^	5.10^5^	0.070
*Vibrio splendidus*	3.10^5^	3.10^6^	0.050	9.10^3^	4.10^5^	0.069
*Vibrio ordalii*	5.10^5^	7.10^6^	0.046	9.10^3^	4.10^5^	0.085
*Vibrio tubiashii*	10^5^	5.10^5^	0.116	7.10^3^	4.10^4^	0.122

The manufacturer's positive control, composed of *V. cholerae, V. vulnificus, V. parahaemolyticus* lyophilised cell extracts, demonstrated a LOD of 8×10^4^ cells ml^−1^ along with dramatically higher absorbance signals (Figure S1). The increased affinity of the antibodies for our strain of *V. parahaemolyticus* (Figure 2) is likely the result of this species being used in the development of the polyclonal antibodies as stated by the manufacturer.

### Immobilisation of the capture antibody onto the neutravidin layer

To enhance specificity, immobilisation of the capture antibody using a neutravidin coated well surface was assessed. Neutravidin is a modified form of avidin from which carbohydrates have been removed, which results in lowering the protein isoelectric point (from 10.5 to 6.3), leading to a decrease in the number of positive charges at neutral pH [32] and thus limited non-specific binding. Effective capture and detection of *V. parahaemolyticus* cells was obtained with concentrations of 20 µg mL^−1^ of Bt-αVib Pab and above. This indicated successful formation of a covalent bond and proper orientation of the antibodies for the capture (Figure S2). An antibody concentration of 20 µg mL^−1^ was sufficient to provide an effective coverage of the neutravidin layer. Using higher concentrations did not result in an increase of the capture efficiency and would only result in a rise of the functionalisation process costs. These results are consistent with those obtained in a previous study using other biotinylated antibodies [31].

Consistently, we did not observe significant levels of bacterial non-specific adsorption on neutravidin-coated surfaces (Figure S2), while avidin has been repeatedly reported to promote binding of negatively charged components, such as nucleic acids and cells [33]–[35]. When no neutravidin was present and thus the Bt-αVib Pab was directly adsorbed on the surface, the bacterial cells were either not detected or very poorly detected even when using a high concentration of antibodies (Figure S2; dashed line). The optimal time and temperature of incubation necessary to facilitate the bonding between the neutravidin and biotinylated molecules (37°C for 1 hour) had been determined in a previous study [31].

### Optimal conditions for *Vibrio* cell capture and detection

To reduce costs for a future *Vibrio* biosensor and limit non-specific adsorption, dilutions of the HRP-α*Vib* Pab ranging from 1/500 to 1/10000 were conducted to determine the lowest effective concentration for detection. Dilutions of 1/2500 and higher affected the assay sensitivity, not allowing the detection to occur (Figure S3a). The LODs obtained from 1/1000 and 1/500 dilutions were the lowest, showing similar values of 8×10^3^ cells mL^−1^ (Figure S3a). However, the non-specific binding of the antibody increased significantly when the 1/500 dilution was used. To limit the non-specific signal, the 1/1000 dilution was considered optimal.

In an attempt to reduce assay time and develop a simplified protocol for the development of a potential *Vibrio* biosensor, the HRP-α*Vib* Pab and the target cells were incubated together for 1 hour at 37°C prior to capture them onto the functionalised surface, thus removing a washing step. However, the efficiency of bacterial capture and detection was dramatically reduced (Figure S3b). This loss in the capture and detection efficiency is likely due to a steric constraint due to excessive cell coverage by HRP-α*Vib* Pab which does not allow further capture onto the functionalised surface, thus this step is not recommended in the final protocol.

Optimised incubation times for both the capture (binding of cells onto functionalised well surfaces) and detection (binding of HRP labelled antibody onto cells) steps were determined to be 30 minutes (see Figure S4). No improvement of the LOD was observed with incubation times longer than 30 min with the HRP-α*Vib* Pab (Figure S4c), which is favourable for developing an assay due to the short time-to-result. Incubation periods shorter than 30 min considerably affected both the capture and detection efficiency.

**Figure 3:**
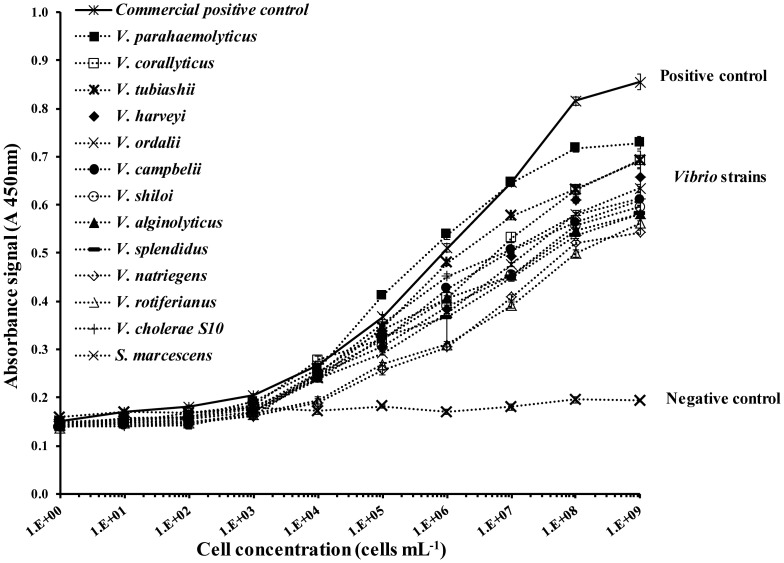
Affinity of the different *Vibrio* strains and commercial positive control to the functionalised surface using optimised conditions. Absorbance signals obtained after a 30 min cell capture step on the functionalised surface and a 30 min detection step using a 1/1000 dilution of horseradish peroxidase anti-*Vibrio* antibody (HRP-α*Vib* Pab).

An affinity test using optimised conditions demonstrated an even distribution of the detection curve for the different *Vibrio* strains and confirmed that the negative control did not bind with the antibody (Figure 3). The results for the *Vibrio* strains and the positive control were more similar compared to the initial experiment when the well plates were not functionalised (Figure 2) and thus functionalisation of the well plate surface was critical to the assay development. Potentially, the high proportion of cell debris contained in the lyophilised positive control resulted in an increased amount of antigen binding onto the bare well surfaces in the initial experiment compared to the specific capture provided by the functionalised surface. Additionally, the amplitude of the signals obtained for the *Vibrio* strains tested were higher due to the enhanced capture capacity of the surface for entire cells.

The LOD, IC50 and sensitivity values obtained when using the functionalisation strategy considerably improved for each strain tested (Table 2). The LOD obtained for all twelve strains tested in this study ranged between 7×10^3^ to 3×10^4^ cells mL^−1^ which represents an improvement of over one order of magnitude compared to the method involving direct adsorption presented in the preliminary experiment (Table 2).

### Detection of *Vibrios* in seawater

**Figure 4:**
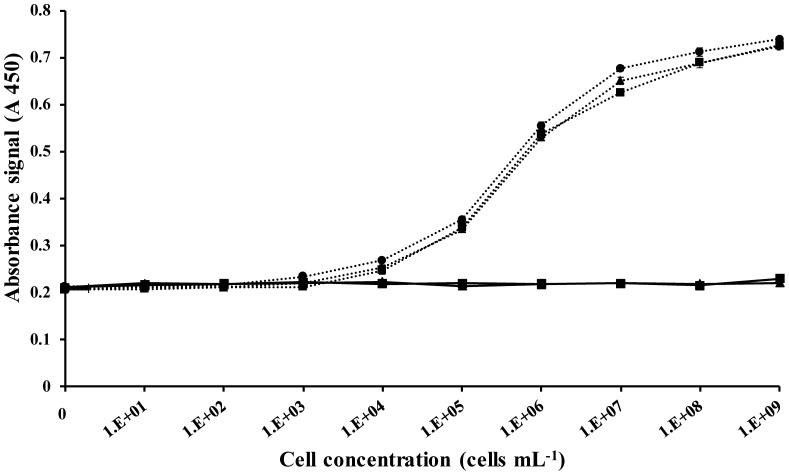
Effect of the capture medium. Signals obtained for increasing *Vibrio parahaemolyticus* (dashed line) or *S. marcescens* (solid line) cell concentrations when carrying out the capture step either in PBS (▪), filtered (▴) or unfiltered (•) natural seawater.

To assess whether the physical and chemical properties of seawater had an antagonistic effect on the capture and detection of the bacterial cells, the sensitivity of the assay was tested in an environmentally realistic sample matrix. We inoculated PBS, 0.2 µm filtered and unfiltered seawater collected from Iron Cove, Sydney, with *V. parahaemolyticus* and *S. marcescens* separately. Similar results were obtained for all sample types (Figure 4) with no visible negative effect of the seawater matrix on the test efficiency and no increase of the non-specific signal. When inoculating filtered seawater or PBS with increasing concentrations of *V. parahaemolyticus*, the LOD remained constant for both media at 7×10^3^ cells mL^−1^ which shows the applicability of the assay developed to environmental samples.

Interestingly, when using unfiltered seawater to prepare our serial dilution of *V. parahaemolyticus* cells, the results showed a slightly lower LOD at 6×10^3^ cells mL^−1^. An enumeration of total *Vibrios* using TCBS agar plate culture revealed a conservative *Vibrio* estimate of 5×10^2^ cells mL^−1^ in the collected sample. This would explain this increase in the detection signal of the assay as we are also detecting the Vibrios already present in the sampled water.

### Detection of *Vibrios* in mulloway fish rearing tanks

**Figure 5:**
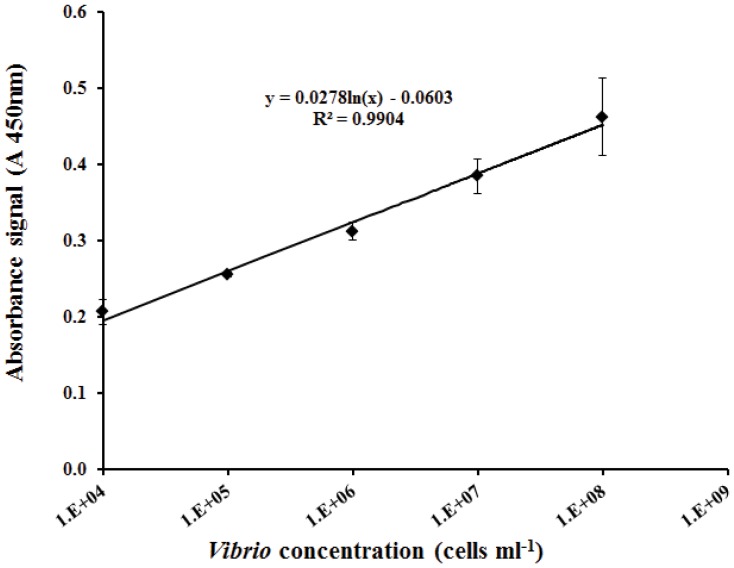
Linear part of the ELISA standard curve. Absorbance values obtained for increasing *V. parahaemolyticus* cell concentrations ranging from 1×10^4^ to 1×10^8^ cells ml^−1^.

A standard curve obtained with the ELISA method using cultured *V. parahaemolyticus* showed a linear relationship between Absorbance and cell abundance between 1×10^4^ and 1×10^8^ cells ml^−1^) with an R^2^ of 0.9904 (p<0.05; Figure 5). The equation fitting the values obtained for this region of the standard curve was used to calculate the *Vibrio* concentrations within mulloway tank samples when samples showed absorbance values above the limit of detection. The comparative estimates of total *Vibrio* abundance in the tank samples using both culturing or ELISA detection methods are shown in Table 3. When within the limit of detection, the counts obtained from the ELISA method showed good correspondence with those obtained using the TCBS plate culture method, but those for the ELISA were slightly higher, most likely because this technique enables the detection of total *Vibrio* cells and not only cultivable cells, and because the culture methods only estimate cells able to grow on the medium. These results confirm that the ELISA method can be used as a reliable warning system when *Vibrio* bacteria concentrations reach values above 10^4^ cells ml^−1^ in aquaculture tanks, providing results within 70 min after sampling.

**Table 3:** Comparison of the *Vibrio* counts obtained using both the TCBS plate culturing and ELISA detection method.

Treatment	Tank	Total bacteria counts obtained from marine agar plates (CFU mL^−1^)	Total *Vibrio* counts obtained from TCBS agar plates (CFU mL^−1^)	Total *Vibrio* counts obtained from the ELISA detection method (Cells mL^−1^)
Clay	1	1.3×10^5^	9.3×10^2^	Below limit of detection
	2	1.5×10^5^	1×10^3^	Below limit of detection
	3	3.8×10^4^	4.5×10^3^	Below limit of detection
Algae	1	4.8×10^4^	1.5×10^4^	3.08×10^4^
	2	1.2×10^5^	3.0×10^4^	3.66×10^4^
	3	3×10^4^	2.3×10^4^	3.98×10^4^

## Conclusions

This study developed a robust surface functionalisation strategy for the capture and colorimetric detection of *Vibrio* cells, and represents an important step for the monitoring of emerging *Vibrio*-related diseases in industrial facilities such as aquaculture systems and the marine environment. The results confirm the efficiency of the functionalisation method by anchoring biotinylated PAb to a neutravidin coated surface and that non-specific adsorption of cells was avoided with the BSA blocking step. No significant cross reactivity was found against the control bacterium tested using this commercial antibody. The assay enables the detection of *Vibrio* cells in seawater samples within 70 minutes, with the LOD ranging between 7×10^3^ to 3×10^4^ cells mL^−1^ without the need for a prior enrichment step. While these LOD are considerably higher than those obtained using other methods such as quantitative PCR which can allow detection of single cells in a sample, they are acceptable for environmental detection and monitoring in aquaculture systems as 1×10^4^ *Vibrio* cells mL^−1^ is standardly present in these environments [2], [4], [36] with the infective dose ranging between 10^4^ and 10^8^ cells mL^−1^ [37]. Therefore, this assay has a significant potential for the rapid detection of *Vibrio* outbreaks in marine systems providing a cost effective, short time-to-result test.

Work is underway to develop the ELISA assay further, and an electrochemical detection format has been developed using this functionalization strategy to immobilise the antibodies onto a gold screen-printed electrode. The secondary antibodies were then detected using amperometric detection of the HRP label [38]. Such a tool contributes significantly to further reducing the LOD obtained in this study, allowing a future miniaturisation and automation of a *Vibrio* online monitoring system. Another development of this method would involve using antibodies which are specific to particular *Vibrio* species of interest for the aquaculture industry in order to detect specific emerging pathogenic Vibrios in rearing facilities. This strategy has recently been used for the screening of *V. Harveyi* in shrimps [39]. These developments will allow us to detect Vibrios rapidly and thus help improve aquaculture yields and provide intervention opportunities if outbreaks occur.

## Supporting Information

Figure S1 **Antibody affinity for different** ***Vibrio*** **strains.** Absorbance signals obtained after direct adsorption of the commercial positive control cells to the well surface for one hour followed by detection using a 1/500 dilution of horseradish peroxidase anti-*Vibrio* antibody (HRP-α*Vib* Pab) incubated for one hour to allow binding onto the surface.
(TIF)

Figure S2 **Biotinylated anti-** ***Vibrio*** **antibody (Bt-α** ***Vib*** **Pab) concentration optimization.** Signals obtained after wells pre-coated with 20 µg mL^−1^ neutravidin (solid lines) and functionalised with increasing concentrations of Bt-α*Vib* Pab were exposed to different concentrations of *Vibrio parahaemolyticus*. Wells in which a 80 µg mL^−1^ Bt-α*Vib* Pab solution was added when no neutravidin was present were also exposed to *V. parahaemolyticus* (dashed line). Bt-α*Vib* Pab concentrations tested were (⧫) 0, (▵) 10, (▪) 20, (□) 40 and (▴) 80 µg mL^−1^. A 1/500 dilution of horseradish peroxidase anti-*Vibrio* antibody (HRP-α*Vib* Pab) was used for the detection of the captured cells.
(TIF)

Figure S3 **Horseradish peroxidase anti-** ***Vibrio*** **antibody (HRP-α** ***Vib*** **Pab) concentration optimization.** Signals obtained after wells pre-coated with 20 µg mL^−1^ neutravidin and functionalised with 20 µg mL^−1^ biotinylated anti-*Vibrio* antibody (Bt-α*Vib* Pab) were exposed to different concentrations of *Vibrio parahaemolyticus* and detected using increasing HRP-α*Vib* Pab dilutions: (▪) 1/500, (□) 1/1000, (•) 1/2500, (○) 1/5000 and (▴) 1/10000. (A) The cells and the HRP-α*Vib* Pab were incubated successively onto the functionalised surface or (B) both the cells and the HRP-α*Vib* Pab were incubated together, before being placed in contact with the surface.
(TIF)

Figure S4 **Capture and detection time optimisation.** Absorbance signals obtained after exposure of wells pre-coated with 20 µg mL^−1^ neutravidin and functionalised with 20 µg mL^−1^ biotinylated anti-*Vibrio* antibody (Bt-α*Vib* Pab) to different concentrations of *Vibrio parahaemolyticus* using increasing contact time with the cells ((A) 5, (B) 10, (C) 30 and (D) 60 mins) and the 1/1000 horseradish peroxidase anti-*Vibrio* antibody (HRP-α*Vib* Pab): (▪) 5 mins, (○) 30 mins and (•) 60 mins.
(TIF)

## References

[1] Pfeffer CS, Hite MF, Oliver JD (2003) Ecology of *Vibrio vulnificus* in estuarine waters of eastern North Carolina. Appl Env Microbiol 69 (6) 3526–3531. 12788759 10.1128/AEM.69.6.3526-3531.2003 PMC161514

[2] Leytoon Y, Riquelme C (2008) *Vibrios* in the marine coastal systems. Rev Biol Mar Oceanog 43 (3) 441–456.

[3] Oberbeckmann S, Wichels A, Maier T, Kostrzewa M, Raffelberg S, et al (2011) A polyphasic approach for the differentiation of environmental *Vibrio* isolates from temperate waters. FEMS Microbial Ecol 75: 145–162. 10.1111/j.1574-6941.2010.00998.x 21118277

[4] Elston RA, Hasegawa H, Humphrey KL, Polyak IK, Hase C (2008) Re-emergence of *Vibrio tubiashii* in bivalve shellfish aquaculture: severity, environmental drivers, geographic extent and management. Dis Aqua Org 82: 119–134. 10.3354/dao01982 19149375

[5] Ralston EP, Kite-Powell H, Beet A (2011) An estimate of the cost of acute health effects from food and water-borne marine pathogens and toxins in the USA. J Wat Health 9: 680–694. 10.2166/wh.2011.157 PMC5439350 22048428

[6] Subasinghe RP, Bondad-Reantaso MG, McGladdery SE (2001) Aquaculture development, health and wealth. In: Aquaculture in the Third Millennium. Technical Proceedings of the Conference on Aquaculture in the Third Millennium ed., Subasinghe R.P., Bueno P., Phillips M.J., Hough C., McGladdery S.E., et al.., (Eds.) Rome, Italy: NACA, Bangkok and FAO, 167–191.

[7] Morris JG (2003) Cholera and other types of vibriosis: a story of human pandemics and oysters on the half shell. Clin Infect Dis 37: 272–280. 12856219 10.1086/375600

[8] Traore SG, Bonfoh B, Krabi R, Odermatt P, Utzinger J, et al (2012) Risk of *Vibrio* transmission linked to the consumption of crustaceans in coastal towns of Cote d'ivoire. J Food Protec 75 (6) 1004–1011. 10.4315/0362-028X.JFP-11-472 PMC4825881 22691466

[9] Oliver JD (2005) Wound infections caused by *Vibrio vulnificus* and other marine bacteria. Epidem Infect 133: 383–391. 10.1017/s0950268805003894 PMC2870261 15962544

[10] Yu W-T, Jong K-J, Lin Y-R, Tsai S, Tey YH, et al (2013) Prevalence of *Vibrio parahaemolyticus* in oyster and clam culturing environments in Taiwan. International J Food Microbiol 160: 185–192. 10.1016/j.ijfoodmicro.2012.11.002 23290223

[11] Senderovich Y, Izhaki I, Halpern M (2012) Fish reservoirs and vectors of *Vibrio cholerae* . Plos ONE 5 (1) 10.1371/journal.pone.0008607 PMC2797615 20066040

[12] Sainz-Hernandez JC, Maeda-Martinez AN (2005) Sources of *Vibrio* bacteria in mollusc hatcheries and control methods: a case study. Aquaculture Res 36: 1611–1618.

[13] Kimes NE, Grim CJ, Johnson WR, Hasan NA, Tall BD, et al (2012) Temperature regulation of virulence factors in the pathogen *Vibrio coralliilyticus* . The ISME J 6: 835–846. 22158392 10.1038/ismej.2011.154 PMC3309362

[14] Vezzulli L, Brettar I, Pezzati E, Reid PC, Colwell RR, et al (2012) Long-term effects of ocean warming on the prokariotic community: evidence from the *vibrios* . The ISME J 6: 21–30. 21753799 10.1038/ismej.2011.89 PMC3246245

[15] Nogales B, Lanfranconi MP, Pina-Villalonga JM, Bosh R (2011) Anthropogenic perturbations in marine microbial communities. FEMS Microbiol Rev 35: 275–298. 20738403 10.1111/j.1574-6976.2010.00248.x

[16] Niemi G, Wardrop D, Brooks R, Anderson S, Brady V, et al (2004) Rationale for a new generation of indicators for coastal waters. Environ Health Perspect 112 (9) 979–985. 15198917 10.1289/ehp.6903 PMC1247190

[17] Sherman K, Aquarone MC, Adams S (eds) (2009) Sustaining the World's Large Marine Ecosystems. Gland, Switzerland: IUCN.

[18] Zhao X, Wang L, Chu J, Li Y, Li Y, et al (2010) Rapid detection of *Vibrio parahaemolyticus* strains and virulent factors by loop-mediated isothermal amplification assays. Food Science Biotech 19 (5) 1191–1197.

[19] Nordstrom JL, Vickery CL, Blackstone GM, Murray SL, DePaola A (2007) Development of a multiplex real-time PCR assay with an internal amplification control for the detection of total and pathogenic *Vibrio parahaemolyticus* bacteria in oysters. Appl Environ Microbiol 73 (18) 5840–5847. 17644647 10.1128/AEM.00460-07 PMC2074920

[20] Jones JL, Hara-Kudo Y, Krantz JA, Benner RA Jr, Smith AB, et al (2012) Comparison of molecular detection methods for *Vibrio parahaemolyticus* and *Vibrio vulnificus* . Food Microbiol 30: 105–111. 22265290 10.1016/j.fm.2011.12.011

[21] Rao VK, Sharma MK, Goel AK, Singh L, Sekhar K (2006) Amperometric immunosensor for the detection of *Vibrio cholerae O1* using disposable screen-printed electrodes. Anal Science 22: 1207–1211. 10.2116/analsci.22.1207 16966811

[22] Jyoung J-Y, Hong S, Lee W, Choi J-W (2006) Immunosensor for the detection of *Vibrio cholerae O1* using surface plasmon resonance. Bios Bioelec 21: 2315–2319. 10.1016/j.bios.2005.10.015 16326089

[23] Barker D, Allan GL, Rowland SJ, Pickles JM (2002). “A Guide to Acceptable Procedures and Practices for Aquaculture and Fisheries Research”. 2 nd edn.

[24] Fielder DS, Heasman MP (2011) Hatchery Manual for the production of Australian Bass, Mulloway and Yellowtail Kingfish. Industry & Investment NSW, ISBN 978 1 74256 058 8. 176 pp.

[25] Boucher Y, Nesbo C, Joss M, Robinson A, Mabbutt B, et al (2006) Recovery and evolutionary analysis of complete integron gene cassette arrays from *Vibrio* . BMC Evol Biol 6 (1) 3. 16417647 10.1186/1471-2148-6-3 PMC1382264

[26] Thompson CC, Vicente AC, Souza RC, Vasconcelos AT, Vesth T, et al (2009) Genomic taxonomy of *Vibrios* . BMC Evol Biol 9: 258. 19860885 10.1186/1471-2148-9-258 PMC2777879

[27] Islam A, Labbate M, Djordjevic SP, Alam M, Darling A, et al (2013) Indigenous *Vibrio cholerae* strains from a non-endemic region are pathogenic. Open Biol 3 (2) 120181. 23407641 10.1098/rsob.120181 PMC3603452

[28] Chatterjee S, Haldar S (2012) *Vibrio* related diseases in aquaculture and development of rapid and accurate identification methods. Journal of Marine Science Research and Development S1: 002.

[29] Pfeffer C, Oliver JD (2003) A comparison of thiosulphate-citrate-bile salts-sucrose (TCBS) agar and thiosulphate-chloride-iodide (TCI) agar for the isolation of Vibrio species from estuarine environments. Let Appl Microbiol 36 (3) 150–151. 12581373 10.1046/j.1472-765x.2003.01280.x

[30] Porter JW, Lipp EK, Sutherland KP, Mueller E (2008) The ecology of an infectious coral disease in the Floride keys: from pathogens to politics, in: Ostfeld RS, Keesing F, Eviner VT (Eds.), Infectious disease ecology: effects of ecosystems on disease and of disease on ecosystems. Princeton university press, pp. 387–403.

[31] Laczka O, Baldrich E, Del Campo FJ, Munoz FX (2008) Immunofunctionalisation of gold transducers for bacterial detection by physisorption. Anal Bioanal Chem 391: 2825–2835. 18553187 10.1007/s00216-008-2199-1

[32] Polysciences Inc (2008) The Benefits of Neutravidin over Streptavidin and Avidin. Polysciences, Inc, Warrington. PA Technical data sheet 779.

[33] Wood GS, Warnke R (1981) Suppression of Endogenous Avidin-Binding Activity in Tissues and Its Relevance to Biotin-Avidin Detection Systems. J Histochem Cytochem 29 (10) 1196–1204. 7028859 10.1177/29.10.7028859

[34] Green NM (1975) Avidin. Adv Prot Chem 29: 85–133. 10.1016/s0065-3233(08)60411-8 237414

[35] Green NM (1990) Avidin and streptavidin. Met Enzymol 184: 51–67. 10.1016/0076-6879(90)84259-j 2388586

[36] Eiler H, Pernthaler J, Aman R (2000) Succession of pelagic marine bacteria during enrichment: a close look at cultivation-induced shifts. Appl Environ Microbiol 66 (11) 4634–4640. 11055904 10.1128/aem.66.11.4634-4640.2000 PMC92360

[37] Percival S, Chalmers R, Embrey M, Hunter P, Sellwood J, et al.. (2004) Microbiology of waterborne diseases. Elsevier academic press, London.

[38] Laczka O, Labbate M, Doblin M (2014) Anal. Methods 6 (7) 2020–2023.

[39] Buchatipa S, Ananthanawat C, Sithigornguld P, Sangvaniche P, Rengpipat S, et al (2009) Detection of the shrimp pathogenic bacteria, Vibrio harveyi, by a quartz crystal microbalance-specific antibody based sensor. Sensors and Actuators B: Chemical 145: 259–264.

