# Supplementary figures and images for: Surface Immuno-Functionalisation for the Capture and Detection of Vibrio Species in the Marine Environment: A New Management Tool for Industrial Facilities
Source: PLoS One. 2014 Oct 13;9(10):e108387. doi: 10.1371/journal.pone.0108387 (PMC4195594; doi:10.1371/journal.pone.0108387)

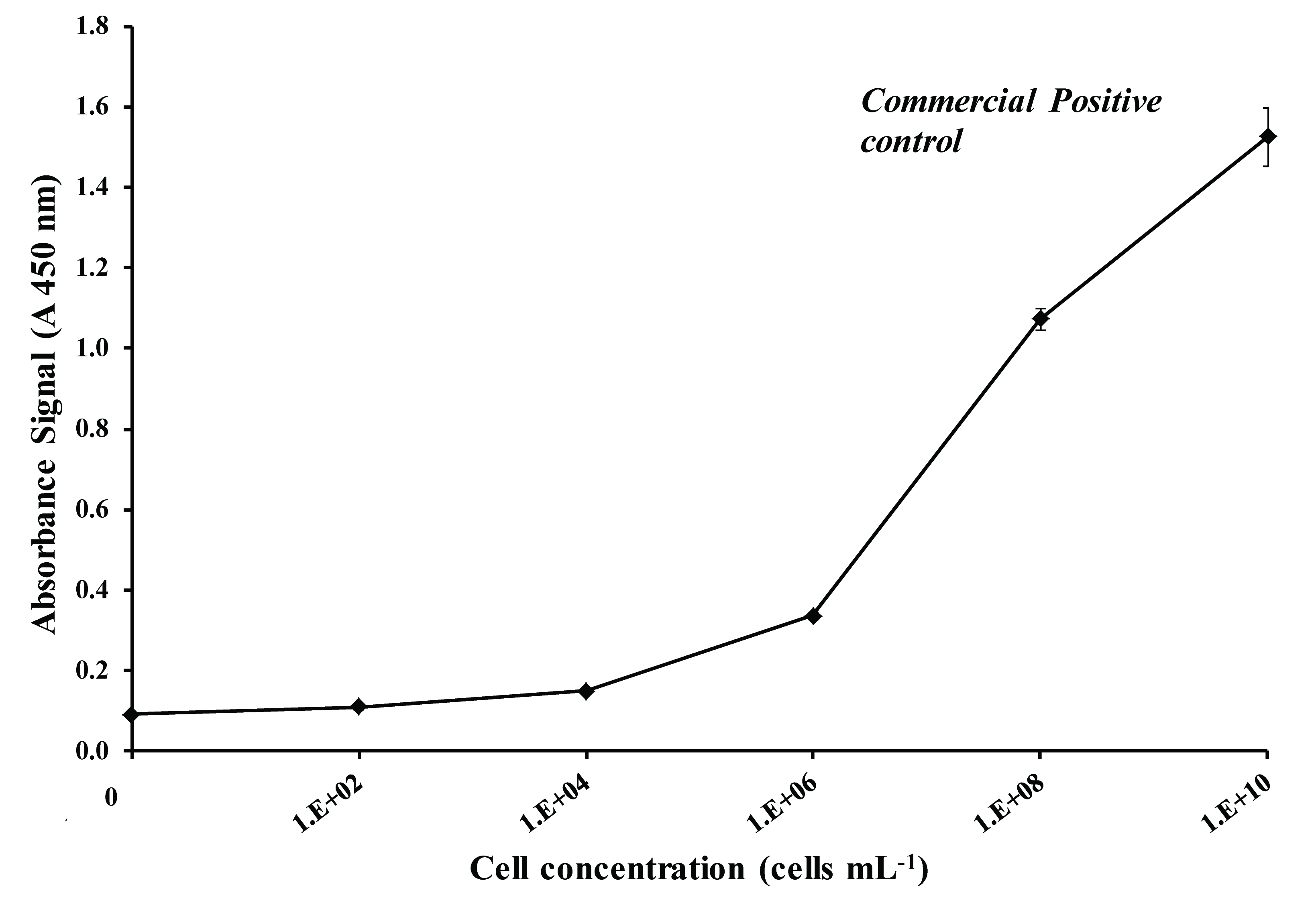

Supplement: Figure S1 — Antibody affinity for different Vibrio strains. Absorbance signals obtained after direct adsorption of the commercial positive control cells to the well surface for one hour followed by detection using a 1/500 dilution of horseradish peroxidase anti-Vibrio antibody (HRP-αVib Pab) incubated for one hour to allow binding onto the surface. (TIF) [file pone.0108387.s001.tif]

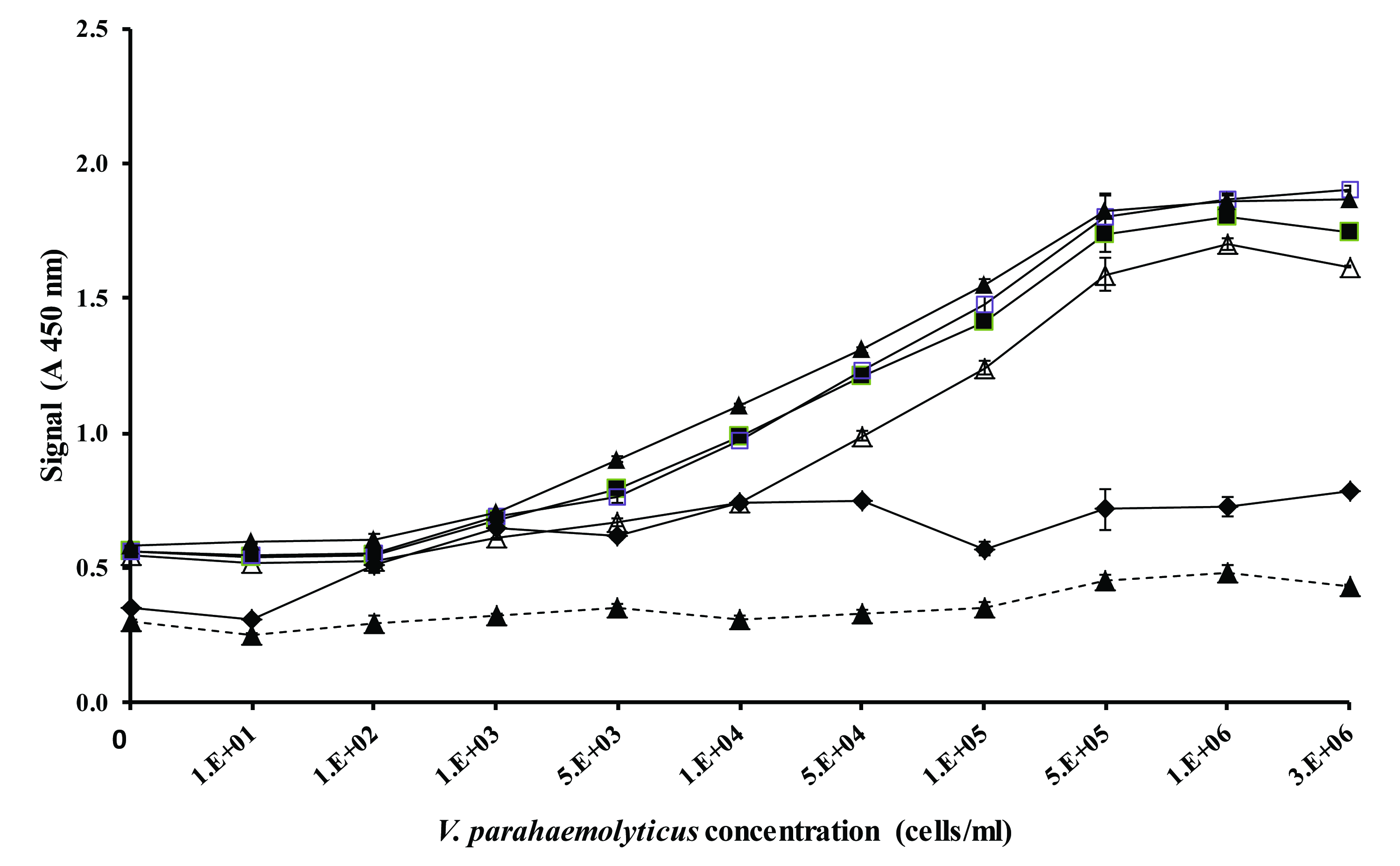

Supplement: Figure S2 — Biotinylated anti- Vibrio antibody (Bt-α Vib Pab) concentration optimization. Signals obtained after wells pre-coated with 20 µg mL−1 neutravidin (solid lines) and functionalised with increasing concentrations of Bt-αVib Pab were exposed to different concentrations of Vibrio parahaemolyticus. Wells in which a 80 µg mL−1 Bt-αVib Pab solution was added when no neutravidin was present were also exposed to V. parahaemolyticus (dashed line). Bt-αVib Pab concentrations tested were (⧫) 0, (▵) 10, (▪) 20, (□) 40 and (▴) 80 µg mL−1. A 1/500 dilution of horseradish peroxidase anti-Vibrio antibody (HRP-αVib Pab) was used for the detection of the captured cells. (TIF) [file pone.0108387.s002.tif]

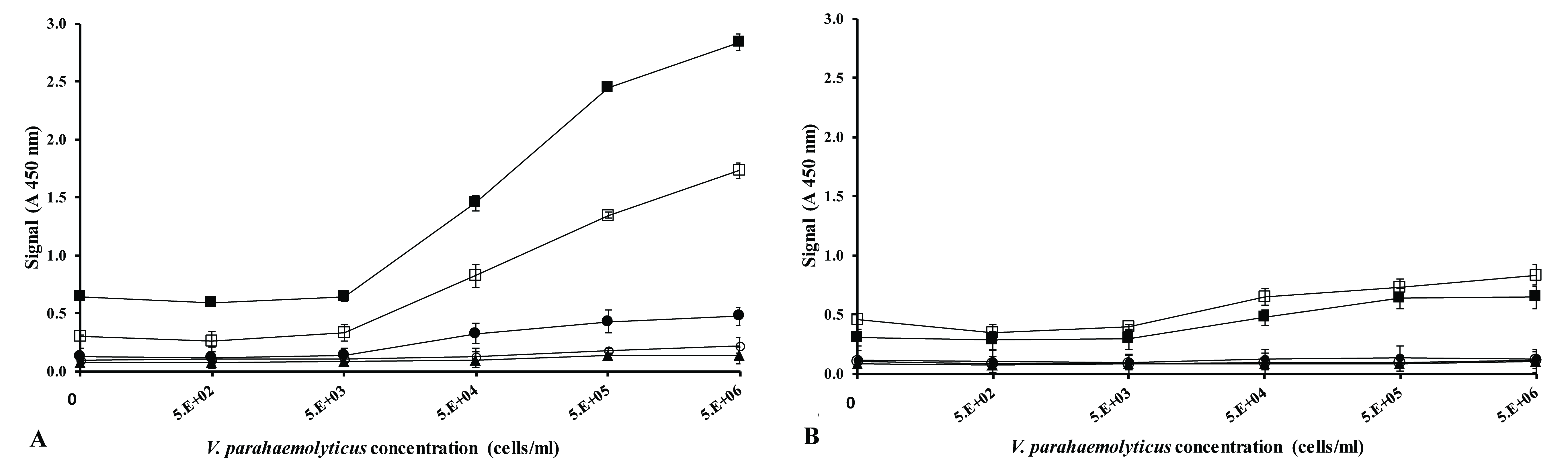

Supplement: Figure S3 — Horseradish peroxidase anti- Vibrio antibody (HRP-α Vib Pab) concentration optimization. Signals obtained after wells pre-coated with 20 µg mL−1 neutravidin and functionalised with 20 µg mL−1 biotinylated anti-Vibrio antibody (Bt-αVib Pab) were exposed to different concentrations of Vibrio parahaemolyticus and detected using increasing HRP-αVib Pab dilutions: (▪) 1/500, (□) 1/1000, (•) 1/2500, (○) 1/5000 and (▴) 1/10000. (A) The cells and the HRP-αVib Pab were incubated successively onto the functionalised surface or (B) both the cells and the HRP-αVib Pab were incubated together, before being placed in contact with the surface. (TIF) [file pone.0108387.s003.tif]
